# Cardiopulmonary baroreceptors modify pain intensity in patients with chronic back pain

**DOI:** 10.21203/rs.3.rs-3154622/v1

**Published:** 2023-07-17

**Authors:** Yuto Iwakuma, Davina A. Clonch, Jennifer Liu, Christopher M. Lam, Seth Holwerda

**Affiliations:** University of Kansas Medical Center; University of Kansas Medical Center; KCU: Kansas City University; University of Kansas Medical Center; University of Kansas Medical Center

**Keywords:** Baroreflex, chronic back pain, habituation, hypertension, cardiopulmonary baroreceptors, neuropathic pain

## Abstract

**Objective:**

Baroreceptors play a significant role in nociceptive pain. However, the extent to which baroreceptors modulate nociception in patients with chronic pain is unclear. We tested the hypothesis that cardiopulmonary baroreceptor unloading via LBNP would significantly increase pressure pain threshold and habituation to heat pain among patients with chronic back pain.

**Methods:**

Mechanical pressure pain threshold at the upper trapezius (hand-held algometer) and habituation to heat pain at the forearm were performed during sitting and supine position, and during baroreceptor unloading via lower body negative pressure (LBNP) of −10 mmHg in 12 patients with chronic back pain (54 ± 11 years of age). To determine whether pain reduction is normal during LBNP, studies were repeated in 7 young, healthy participants (23 ± 7).

**Results:**

Mechanical pressure pain threshold (P < 0.01) and habituation to heat pain (P = 0.04) were significantly reduced during supine compared with sitting. Conversely, baroreceptor unloading via LBNP significantly increased pressure pain threshold (P = 0.03) and heat pain habituation (P < 0.01) compared with supine. In young healthy controls, pressure pain threshold was similarly affected when comparing sitting and supine (P = 0.01) and during LBNP (P < 0.01), whereas habituation to heat pain was unaltered when comparing sitting and supine (P = 0.93) and during LBNP (P = 0.90). Total peripheral resistance was increased during LBNP (P = 0.01) but not among young, healthy controls (P = 0.71).

**Conclusions:**

The findings demonstrate cardiopulmonary baroreceptor modulation of nociceptive pain in patients with chronic pain. Interestingly, habituation to heat pain appears more readily modified by cardiopulmonary baroreceptors in patients with chronic back pain compared with young, healthy individuals.

## INTRODUCTION

Chronic pain is one of the most common reasons for clinic visits in the United States with an annual cost estimated to be nearly $635 billion per year [1]. Patients with chronic pain often display alterations in the autonomic nervous system [2], including impairment in cardiovascular autonomic function [3]. Associations have been identified between cardiovascular autonomic parameters and brain regions involved with nociceptive information [4]. Indeed, altered baroreceptor function has been linked to maintenance of chronic pain [5]. However, the extent to which cardiovascular autonomic activity modulates nociceptive information in chronic pain remains uncertain.

Pain perception depends on activation of the descending pain modulatory circuit. Spinal or medullary dorsal horns receive descending input originating from numerous brain regions, such as the hypothalamus, amygdala, the rostral anterior cingulate cortex, and the periaqueductal grey to enhance or diminish nociceptive transmission [6–8]. Descending pain inhibition, for example, may cause a reduction in individual responses to the same painful stimulus (i.e., habituation). Reduced or absent habituation has been reported in states of chronic pain, such as chronic back pain [9, 10], fibromyalgia [11–13], and migraine [14]. However, despite changes in pain perception and cardiovascular autonomic function in chronic pain, the understanding of the neural mechanisms involved in descending pain modulation remains incomplete.

Evidence in humans suggests that cardiorespiratory afferents modulate pain perception [15–17]. Cardiorespiratory afferents include baroreceptors, which are myelinated (Aδ) and unmyelinated (C) fibers with nerve terminals in the adventitia of the vessel wall. Baroreceptors increase firing rate in response to mechanical stretch of the vessel wall and are critical for reflex control of BP. Cardiopulmonary baroreceptor activation elicits an increase in pain perception [15, 16, 18–20]. For example, pain perception was increased following passive leg elevation to activate cardiopulmonary baroreceptors [18]. Similarly, higher pain perception was observed during hypervolemia conditions (fluid ingestion) compared with euvolemia conditions [19]. However, not all studies have reported changes in pain perception in response to posture maneuvers [16, 21]. Importantly, studies examining baroreceptor control of pain perception in patients with chronic pain have been sparce. Moreover, few studies have examined whether *unloading* of cardiopulmonary baroreceptors, such as during orthostatic stress, elicits a reduction in pain perception or habituation to a painful stimulus.

Therefore, we examined the extent to which cardiopulmonary baroreceptor activity modulates pain perception in patients with chronic low back pain. Perception of mechanical pressure and heat pain was tested during upright sitting and supine, and test during cardiopulmonary baroreceptor unloading via lower body negative pressure (LBNP). Because cardiopulmonary baroreceptor *loading* is expected to increase pain perception [18, 19], we hypothesized that cardiopulmonary baroreceptor *unloading* via LBNP would significantly increase pressure pain threshold and heat pain habituation, i.e., lower perception of pain, among patients with chronic back pain. LBNP was performed only at low intensity (−10 mmHg) and the rationale was to reduce central venous pressure and elicit significant increases in sympathetic nerve activity (~ 25%) while minimizing major changes in steady-state mean blood pressure (BP) and heart rate [22–24]. Higher intensity LBNP is more likely to reduce mean BP and affect arterial baroreceptors, which also have antinociceptive properties [25] and would confound the interpretation of results.

## MATERIALS

All experimental procedures and protocols conformed to the Declaration of Helsinki and were approved by the Institutional Review Board at the University of Kansas Medical Center (STUDY0014659). Each participant received a verbal and written explanation of the study objectives, measurement techniques, and risks and benefits associated with the investigation prior to providing written informed consent on the initial visit.

### Participants

Patients with chronic back pain (> 3 months) were recruited from clinics at the University of Kansas Medical Center (KUMC). Recruitment was performed primarily via email correspondence. Study enrollment was limited to the sample size needed (n = 12) to attain a power of 0.80 to detect a statistically significant change in the heat pain habituation slope during baroreceptor unloading (LBNP − 10 mmHg vs. 0 mmHg) at the level of α = 0.05 with an effect size of 0.85. Inclusion criteria included men or women of the age 18–79 years with chronic back pain, and exclusion criteria included current diagnosis of cancer, active infection, and unstable blood pressure (BP) in past 3 months (e.g., change in antihypertensive medications). CBP patients with history of hypertension were not excluded (n = 1, documented via average ambulatory 24-hr BP monitoring); however, participants were instructed to refrain from anti-hypertensive medications on the day of study visit and to maintain a normal regimen for all other medications, which are described in Table 1. A group of young, healthy participants without history of chronic pain was included to determine if LBNP-mediated changes in heat pain habituation occur normally or if it is specific to chronic pain. Because LBNP-mediated changes in heat pain habituation could not be elicited in young, healthy participants (see results), enrollment was limited to n = 7.

## Experimental Measurements

### Clinical pain assessments:

Chronic pain severity was determined using the Brief Pain Inventory (BPI-sf), which is one of the most common tools for assessing clinical pain and is valid for patients with CBP [26]. The BPI-sf is a numeric rating scale (0–10) consisting of 12 items that assess severity of pain (current symptoms, symptoms on average, and range of pain intensity) and its impact on daily life (how pain interferes with their general activity, mood, mobility, work, relationships, sleep, and enjoyment of life). Pain severity was also determined using the painDETECT (PD-Q) screening tool, which is suitable for CBP and has high sensitivity (85%) and specificity (80%) [27]. The PD-Q is designed to discriminate between nociceptive and neuropathic components of chronic pain (maximum score: 38, minimum score: −1; scores ≤ 12 indicating low probability of neuropathic pain). Patients with a neuropathic pain component(s) (e.g., radiating pain, allodynia) generally suffer more severely than those without [27]. Pain severity was also assessed with the Brief Pain Inventory (BPI-sf), which is one of the most common tools for assessing clinical pain and is valid for patients with CBP [26]. Pain catastrophizing was assessed with the coping strategies questionnaire-catastrophizing subscale (CSQ-CAT), and functional disability was assessed with the Oswestry Disability Index (ODI) (also named Low Back Pain Disability Questionnaire) where 0%–20% indicates minimal disability, 21%–40% indicates moderate disability, 41%–60% indicates severe disability, and 81%–100% indicates bed-bound or patient is exaggerating symptoms.

### Cardiovascular variables

Heart rate was determined from lead II of a three-lead ECG and beat-to-beat BP was monitored via finger photoplethysmography using the Finapres^®^ NOVA (Finapres Medical Systems, Enschede, the Netherlands). Stroke volume was estimated via the Modelflow method [28], and total peripheral resistance (TPR) was calculated as mean arterial pressure (MAP) divided by the product of stroke volume and heart rate. Absolute values of BP were taken from auscultatory BP at the brachial artery during periods of resting baseline.

### Lower body negative pressure (LBNP)

The lower portion of the participant’s body below the iliac crest was enclosed in a box-like chamber to allow the application of negative pressure to the lower body in the supine position. LBNP was performed at −10 mmHg (low intensity) to reduce central venous pressure (~ 3 mmHg) and elicit significant increases in sympathetic nerve activity (~ 25%) without major changes in steady-state BP and heart rate [22–24].

### Thermal pain stimulus

Thermal stimuli were administered using a Medoc Thermal Sensory Analyzer (TSA-II, Medoc, Inc., Ramat, Isreal), which uses dedicated graphic based software to control the pattern, duration, rate of increase/decrease, and intensity of a thermal stimulus from a 30 × 30 mm Peltier thermistor probe to the nondominant ventral forearm. To reach threshold, the heat stimulus increased at a consistent rate and participants were instructed to press a hand-held button the moment when the intensity of the heat stimulus changed from a comfortable heat to an uncomfortable heat, or when the intensity would be rated as 1 on a scale of 0–10. Therefore, the stimulus intensity that was achieved was above the perception threshold and below the pain tolerance.

### Short-term habituation

**Figure 1:**
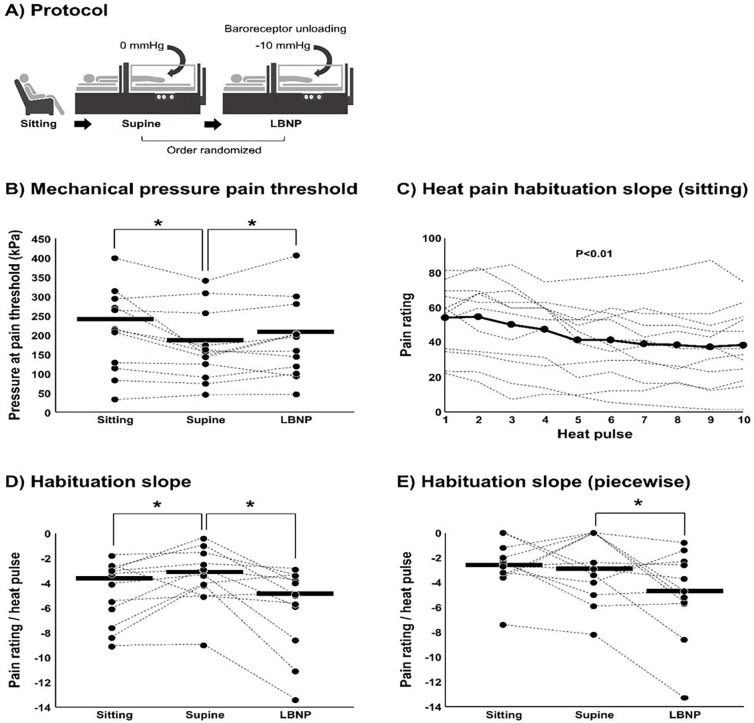
Baroreceptor loading (via posture change from sitting to supine) and baroreceptor unloading via lower body negative pressure (LBNP) was performed in 12 chronic back pain patients (Panel A). Mechanical pressure pain threshold (Panel B) and heat pain habituation (Panels C-E) were performed under each condition. Dotted lines in Panel C represent self-reported pain ratings for heat pulses from each patient and the solid black line represents the mean. The slope of the decline was determined by selecting only consecutive heat pulses where pain ratings declined and excluding all other data (Panel B) and analyzed by using an objective approach (piecewise regression analysis) in which the slope is determined statistically without manually selecting the region where the slope occurs. Because data were not always normally distributed, statistical differences between conditions was determined using Generalized Estimating Equations (GEE). Asterisks indicate significant difference between conditions as indicated (P<0.05).

Short-term habituation was achieved using a repetitive heat stimulus intensity equal to 105% of heat pain threshold (~ 44–46°C). Preliminary studies indicated this stimulus intensity would consistently produce a subjective pain rating of ~ 50 on a scale of 0–100, whereas a stimulus intensity at threshold may result in pain ratings approaching zero by the end of the sequence and therefore “bottom out”. Each sequence of thermal stimuli was programmed to include 10 successive pulses of a 2-s duration and 1-s interval between pulses at a heat intensity equivalent to 105% of the participants’ heat pain threshold delivered from a 40° C baseline (Fig. 1A). The rate of increase and decrease in temperature (temp/sec) was 105% threshold minus baseline (40°C) divided by two (on average ~ 2.0–2.5°C/sec). Participants were asked to rate their perceived pain intensity at the peak of each thermal stimulus on a visual analog scale of 0–100. Importantly, participants were instructed that each successive heat pulse would be somewhat less, more, or the same as their pain threshold intensity in a randomized fashion, and therefore were blinded to the intensity of each heat pulse. However, each heat pulse was in fact programmed to reach the same temperature across the 10 successive pulses (105% of the participants’ heat pain threshold). The probe was moved to different sites on the forearm for each subsequent sequence of pulses.

### Mechanical pressure pain threshold

Pressure pain threshold was assessed using a hand-held mechanical pressure algometer (Algomed) on the left upper trapezius, approximately 2 cm from the acromioclavicular joint. The hand-held algometer is both a reliable and valid process to quantify subjective pain as objective pain [29]. The pressure stimulus increased at a consistent rate for all participants based on the rate graph visualized on the monitor, and participants were instructed to press a hand-held button the moment when the intensity of the pressure stimulus changed from a comfortable pressure to an uncomfortable pressure, or when the intensity would change from a 0 to 1 on a scale of 0–10. Three pressure pain threshold measures were performed at the same anatomical location under each condition (upright sitting, supine, and LBNP − 10 mmHg), and the 3 measurements under each condition were averaged to represent pressure pain threshold.

### Ambulatory 24-hr BP monitoring

Noninvasive 24-hr ambulatory BP, which is regarded as the gold standard for the prediction of risk related to BP [30, 31], was obtained using oscillometric SpaceLabs 90207 monitors (SpaceLabs Healthcare, Snoqualmie, WA) [32]. Monitors were programmed to obtain BP readings at intervals of 30 min during the day from 0600 to 2200 hours and at night every 60 min from 2200 to 0600 hours. At least 10 daytime readings and 5 nighttime readings and at least 80% successful readings of planned measurements over the 24 hours were required [33].

## Experimental protocol

On the first visit to the laboratory, patients received verbal explanation of the study and provided written informed consent. Patients were then familiarized with the computerized pressure algometer to test mechanical pressure pain testing in the upright sitting position (Fig. 1A). Next, participants were familiarized with the LBNP protocol and instrumented for heart rate and beat-to-beat BP (finger photoplethysmography). Testing under each condition (sitting, supine, LBNP) was always preceded by a 5-min quiet rest period. After performing mechanical pressure pain testing during LBNP, chronic back pain patients were instrumented with a 24-hr ambulatory BP monitor to take home and completed clinical pain assessments (self-report questionnaires) via REDCap on a home computer. Within two weeks, participants returned to the laboratory to become familiarized with the computerized thermal stimulator and perform the study protocol of 10 min resting baseline and heat pain testing under sitting, supine, and LBNP conditions. All experiments were performed in a dimly lit room at an ambient temperature of 22–24°C.

### Lower body negative pressure protocol

The lower portion of the participant’s body below the iliac crest was enclosed in a box-like chamber to allow the application of negative pressure to the lower body in the supine position as previously described [34]. Participants underwent a 5 min quiet resting period, then the vacuum motor controlling the chamber pressure was turned on (Fig. 1A). To minimize the potential for cardiovascular responses related to anticipation of LBNP, a countdown was not provided to the participant of when LBNP would begin after the quiet baseline period. The LBNP chamber pressure was reduced at a rate of −0.5 mmHg/s until reaching − 10 mmHg and was continuously monitored. Each participant underwent 4 trials of LBNP (2 min each) and each trial was either 0 mmHg (supine) or −10 mmHg in a randomized fashion separated by at least 5 min of rest. The vacuum motor was on during each trial to provide a consistent auditory stimulus regardless of the condition (supine vs. −10 mmHg). LBNP exposure was limited to 2 min per trial to isolate autonomic from humoral responses [35], and the number of trials were limited to 4 because preliminary studies indicated the supine position in the LBNP chamber may become intolerable over longer duration for some patients with back pain.

## Data analysis

### Short-term heat pain habituation:

The decline in self-reported pain scores is often observed during the first half of the heat pulse sequence (Fig. 1C). Therefore, heat pain habituation was determined by selecting only the declining portion of the sequence and quantifying the slope. The declining portion was determined subjectively and objectively using a segmented (piecewise) linear regression (SegReg, https://www.waterlog.info/segreg.htm), which statistically determines the breakpoints and reports the slope of only the declining portion of the sequence.

### Cardiovascular responses to LBNP

Hemodynamics during LBNP were examined at the onset when chamber pressure began to decrease and then reached − 10 mmHg (30 sec duration), during steady state LBNP at −10 mmHg (and 0 mmHg) when sensory testing was performed (60 sec duration), and during the recovery period after chamber pressure returned to 0 mmHg (120 sec duration). Values for total peripheral resistance, cardiac output, and MAP were calculated as a percent of baseline, which was 2-min immediately preceding the onset of LBNP.

#### Spontaneous cardiac baroreflex sensitivity:

Spontaneous cardiac baroreflex sensitivity was estimated during a 10-min quiet resting period in the supine position using the sequence technique (Nevrokard software, Izola, Slovenia). Briefly, sequences of three or more consecutive beats where systolic BP and R-R interval change in the same direction were identified as baroreflex sequences. A linear regression was applied to each individual sequence, and an overall average was calculated for a measure of spontaneous cardiac baroreflex sensitivity. Only sequences where *R*^2^ was > 0.85 were accepted. Gains were determined for all sequences combined and separately for up (increase systolic BP: increase R-R interval) and down (decrease systolic BP: decrease R-R interval) sequences [36].

### Statistical Analysis

Statistical differences between conditions were tested using Generalized Estimating Equations (GEE) because data were not normally distributed. Data are reported as median with individual data points. Statistical significance was set at *P* < 0.05.

## RESULTS

### Participant characteristics

Patients experienced on average 14 ± 15 years of chronic pain and were on average not hypertensive based on ambulatory 24-hr BP monitoring (Table 1). Two patients declined the ambulatory 24-hr BP cuff because of concern that it would be painful or uncomfortable. The first patient that declined had high average upright sitting BP of 147/92 mmHg during the study visit and the second patient that declined had lower upright sitting BP of 103/70 mmHg.

#### Cardiopulmonary baroreceptors and mechanical pressure pain:

A significant reduction in mechanical pressure pain threshold was observed in the supine position compared with the upright sitting position in chronic back pain patients (P < 0.01) (Fig. 1B). Mechanical pressure pain threshold was significantly increased during cardiopulmonary baroreceptor unloading via LBNP compared with the supine position (P = 0.03) (Fig. 1B). A post hoc analysis on averages and variance (Upright sitting: 240 ± 94 vs. Supine: 202 ± 81 kPa) revealed a strong effect size of 0.90 and power of 0.90. Young, healthy participants without chronic pain demonstrated a similar decrease in mechanical pressure pain threshold when comparing sitting and supine position (Sitting: 259 ± 141 vs. Supine: 179 ± 79 kPa, P = 0.001). Young, healthy participants also showed a similar increase in pressure pain threshold during LBNP (Supine: 179 ± 79 vs. LBNP: 203 ± 88 kPa, P < 0.001).

#### Cardiopulmonary baroreceptors and heat habituation:

In the upright sitting position, a significant reduction in subjective pain ratings (visual analog scale, 0–100) was observed over the ten repetitive heat pulses among chronic back pain patients (P < 0.01), indicating short-term habituation (Fig. 1C). A significant reduction in pain ratings over the ten repetitive heat pulses was also observed in the supine position (P < 0.01) and during LBNP (P < 0.01). However, the slope of the reduction in pain ratings was significantly diminished during supine compared with the upright sitting position (P = 0.04) (Fig. 1D). When slopes were examined objectively via piecewise regression analysis, no significant difference was observed between sitting and supine (P > 0.05) (Fig. 1E). Importantly, when examining cardiopulmonary baroreceptor unloading via LBNP, the slope of the reduction in pain ratings was significantly larger compared with the supine position, regardless of whether slopes were analyzed subjectively (Fig. 1D) or objectively (Fig. 1E) (both P < 0.01). To determine whether it is normal for LBNP to augment heat pain habituation during LBNP, studies were repeated in young, healthy participants without chronic pain. Chronic back pain patients and young, healthy participants demonstrated similar slopes of the reduction in pain ratings when sitting (P = 0.66). However, in contrast to patients with chronic back pain, the slope of the reduction in pain ratings during repetitive heat pain stimuli was not different across conditions among young, healthy participants (Sitting: −4.1 ± 2.5 vs. Supine: −4.2 ± 2.7 vs. LBNP: −3.8 ± 3.3 pain rating·pulse^−1^, P = 0.90).

### Cardiovascular responses to LBNP

**Figure 2:**
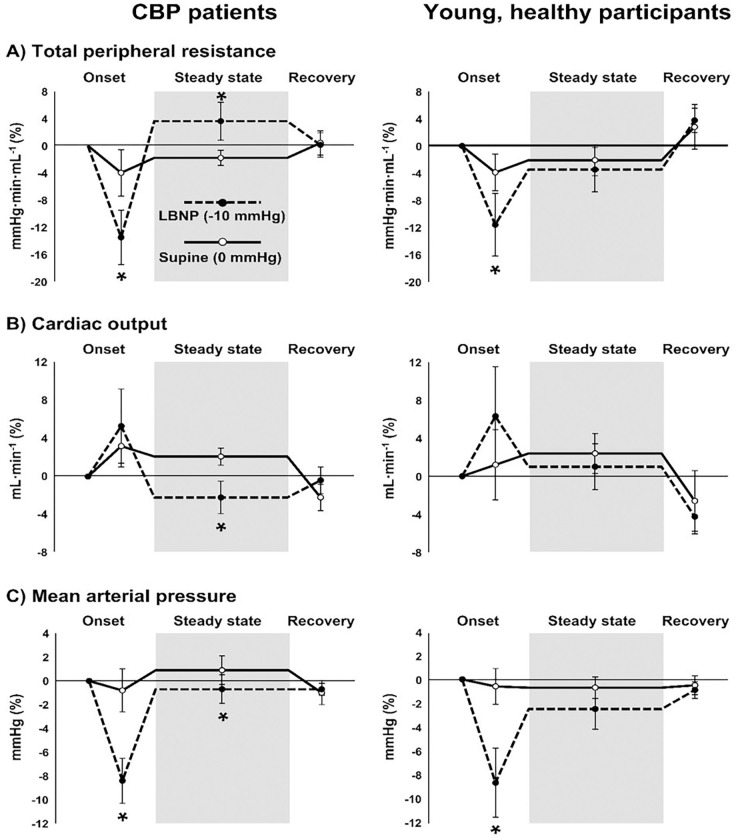
Cardiovascular responses to lower body negative pressure (LBNP). Total peripheral resistance (A), cardiac output (B), and mean arterial pressure (C) during the initial onset of LBNP, during steady state LBNP when sensory testing was performed (shaded region), and during recovery when chamber pressure reaches baseline (0 mmHg) in 12 patients with chronic back pain (left panels) and in 7 young, healthy participants (right panels). Solid lines represent the control condition where no vacuum seal was present and chamber pressure remained at 0 mmHg, and dotted lines represent LBNP with chamber pressure of −10 mmHg (conditions were randomized). Because data were not always normally distributed, differences between timepoints and conditions were tested using Generalized Estimating Equations and pairwise comparisons were performed when significant interactions were detected. * P<0.05 vs. control condition (0 mmHg).

Total peripheral resistance (TPR) and mean arterial pressure (MAP) were significantly reduced during the 30-s onset of LBNP − 10 mmHg compared with the resting supine condition (LBNP 0 mmHg) among chronic back pain patients (Fig. 2, left hand panels). However, during steady-state LBNP when sensory testing was performed (grey region in graph), a significant increase in TPR was observed compared with the control condition (LBNP 0 mmHg) among chronic pain patients (P = 0.01), but not young, healthy controls (P = 0.71) (Fig. 2). Chronic back pain patients also showed a significant reduction in cardiac output (P < 0.01) and MAP (P = 0.02) compared with the control condition (LBNP 0 mmHg). The percent change in HR during LBNP − 10 mmHg was significantly higher among young, healthy participants (7 ± 5 bpm) compared with chronic back pain patients (2 ± 1 bpm) (P = 0.04).

## DISCUSSION

The present study examined cardiopulmonary baroreceptor modulation of pain perception in patients with chronic back pain. Two novel findings are reported. First, cardiopulmonary baroreceptor unloading via LBNP significantly enhanced mechanical pressure pain threshold and enhanced the slope of the reduction in pain ratings of repetitive heat stimuli (heat pain habituation) in patients with chronic back pain. Second, LBNP-mediated changes in heat pain habituation could not be elicited during posture change (supine vs. sitting) or during LBNP in young, healthy participants. Total peripheral resistance was significantly elevated during LBNP compared with the control condition (0 mmHg) among chronic pain patients, but not among young, healthy participants. These findings suggest potential for greater cardiopulmonary baroreceptor sensitivity in chronic back pain patients that may explain, in part, enhanced heat pain habituation during LBNP.

No previous studies, to our knowledge, have directly examined the contribution of cardiopulmonary baroreceptors to pain perception. However, there is evidence for reduced pain perception during significant elevations in sympathetic nerve activity [37]. For example, Dayan et al. [37] reported increased pain adaptation following administration of yohimbine, an α_2_-adrenergic receptor antagonist, in young, healthy participants. Reduced pain perception during elevations in sympathetic nerve activity also do not involve the endogenous opioid system [38]. LBNP performed at −10 mmHg, elicits a significant reflex increase in sympathetic nerve activity of approximately + 25% [22–24] proportional to the reduction in stroke volume [24, 39, 40]. Interestingly, increases in total peripheral resistance were observed among chronic pain patients during LBNP − 10 mmHg, suggesting a reflex-mediated increase in sympathetic outflow, whereas LBNP did not significantly increase total peripheral resistance in young, healthy participants (Fig. 2). Chronic pain patients were significantly older than young, healthy participants (Table 1); however, previous work suggests that advancing age does not increase cardiopulmonary baroreceptor sensitivity during LBNP [41]. Although the present study did not determine cardiopulmonary baroreceptor sensitivity, which is the change in forearm vascular resistance in response to LBNP [42], the results point towards enhanced cardiopulmonary baroreceptor control of heat pain habituation in chronic back pain patients. Further work is needed to confirm whether the magnitude of change in pain perception is positively related to cardiopulmonary baroreceptor sensitivity, and whether cardiopulmonary baroreceptor sensitivity is altered in patients with chronic pain.

Previous studies examining cardiopulmonary baroreceptor control of pain provide evidence that hypertension influences the analgesic response [15, 18, 19]. In the present study, responses to baroreceptor stimulation appear to resemble that of hypertensive patients [15, 18]. For example, lowered sensitivity to heat pain was observed only among borderline hypertensive men during passive leg elevation to load baroreceptors [18]. In the present study, we observed reduced heat pain during baroreceptor loading among chronic pain patients but not young, healthy controls (Fig. 1). Similarly, ratings of a mechanical finger pressure were reduced during passive leg elevation (baroreceptor loading) only among participants with elevated BP [15]. A larger contribution of cardiopulmonary baroreceptors to pain perception in this context may be explained by findings from Mark et al. who showed that cardiopulmonary baroreceptor sensitivity is enhanced using mild lower body negative pressure (e.g., −10 mmHg) in individuals with borderline hypertension [42]. Therefore, it is tempting to speculate that cardiopulmonary baroreceptor sensitivity was higher among chronic pain patients in the present study that resulted in a more robust analgesic response to cardiopulmonary baroreceptor unloading. Although cardiopulmonary baroreceptor sensitivity, i.e., change in forearm vascular resistance in response to LBNP [42], was not determined in the present, increases in total peripheral resistance were observed in response to LBNP − 10 mmHg among chronic pain patients, while no increase in total peripheral resistance was observed among in young, healthy controls (Fig. 2). These findings are indirect evidence that cardiopulmonary baroreceptor sensitivity may be elevated in chronic pain and warrant further studies that include chronic back pain patients with and without established hypertension.

Adaptation to a pain stimulus is dependent on an aggregate of parameters, such as stimulus frequency, intensity, rate of increase/decrease, and anatomical location. Regarding the stimulus frequency, habituation to heat pain has been reported using relatively long duration intervals between stimuli, such as up to 80 sec [43] or 20 sec [44]. When using a short duration interval, such as ≤ 3 sec, sensitization rather than habituation can be observed [45, 46], and the increase in perceived pain during repeated stimuli with intervals of ≤ 3 sec is associated with C nociceptor fiber discharge [43, 47]. However, in the present study, short intervals of 2 sec elicited short-term habituation. Short-term habituation was elicited in the present study because of the lower stimulus intensity. Instead of using a relatively high stimulus intensity that can elicit wind-up (e.g., 53°C) [46], a lower stimulus intensity equal to 105% of heat pain threshold was utilized (~ 44–46°C). These findings are in line with a previous study [44] demonstrating habituation to a heat stimulus when repeatedly testing heat pain threshold in patients with chronic pain and healthy individuals. However, although the interval between stimuli was not reported, it was significantly longer than the present study because each stimuli was a ramp from 32°C to heat pain threshold [44]. Regarding the rate of temperature increase, studies have failed to elicit habituation in chronic pain when using relatively slow rates of temperature increase during stimulation (0.5°C/sec) [12], whereas studies using faster rates of temperature increase successfully elicited habituation (1.5°C/sec) [44]. The present study included a faster rate of temperature increase, which averaged ~ 2.0–2.5°C/sec, depending on the participant’s heat pain threshold. Thus, greater rate of increase in temperature may increase probability for habituation.

There are limitations of the present study. First, adaptation to the heat stimulus is likely body-site-specific and may not occur at a different anatomical site. Therefore, we cannot rule out that heat habituation may not manifest if the heat stimulator was placed at an anatomical site other than the anterior forearm. Second, self-reported pain is not an objective assessment of the pain experience. However, although self-reported pain is used ubiquitously in clinical studies, it is a subjective measure of pain. An example of a more objective measure of pain is the change in the flexion reflex threshold during transcutaneous electrical stimulation of the sural nerve [48, 49], a purely nociceptive nerve that eliminates the reliance on self-reported pain.

In summary, these findings show cardiopulmonary baroreceptor modulation of pain perception in patients with chronic back pain. Findings also suggest a potentially pathophysiological change in cardiopulmonary baroreceptor function in patients with chronic back pain because LBNP did not increase the slope of the change in pain ratings during repetitive heat stimuli (heat pain habituation) in young, healthy participants. Future studies that include modalities of reducing central venous pressure (e.g., diuretics, LBNP) are warranted to confirm our findings in patients with chronic back pain.

## Figures and Tables

**Table 1.** Participant biometrics

	CBP (n=12)	Healthy (n=7)	t-tests
**Age,** years	54 ± 11	23 ± 1	P<0.01
**Chronic pain questionnaires**			
painDETECT	16 ± 10	--	--
BPI-sf	5.5 ± 2.0	--	--
CSQ-CAT	8.8 ± 6.4	--	--
ODI	31 ± 7	--	--
**Chronic pain duration, years**	14 ± 15	--	
**Ambulatory 24-hr BP**			
Avg. systolic BP, mmHg	117 ± 9	--	--
Avg. diastolic BP, mmHg	72 ± 9	--	--
**Resting cardiovascular variables**			
Supine systolic BP, mmHg	128 ± 12	112 ± 12	*P*=0.02
Supine diastolic BP, mmHg	83 ± 9	68 ± 7	*P*<0.01
Sitting systolic BP, mmHg	125 ± 16	113 ± 12	*P*=0.10
Sitting diastolic BP, mmHg	82 ± 10	68 ± 9	*P*<0.01
**Cardiac baroreflex sensitivity**			
BRS up sequences (ms/mmHg)	5.9 ± 4.0	29.5 ± 11.5	*P*<0.01
BRS down sequences (ms/mmHg)	6.6 ± 3.5	27.2 ± 7.6	*P*<0.01
**Medications**			
Prescription opioids, n	4	--	--
Non-steroidal anti-inflammatory, n	8	--	--
Muscle relaxants, n	7	--	--
Anticonvulsants, n	4	--	--
Psychotropic medications, n	9	--	--

Values are means ± SD. BPI-sf, brief pain inventory short form; CSQ-CAT, coping strategies questionnaire-catastrophizing subscale; ODI, Oswestry Disability Index; BP, blood pressure; BRS, spontaneous cardiac baroreflex sensitivity. Comparisons were made using independent t-tests.

## References

[1] Gaskin DJ, Richard P (2012) The economic costs of pain in the United States. J Pain 13:715–724 22607834 10.1016/j.jpain.2012.03.009

[2] Yeater TD, Clark DJ, Hoyos L, Valdes-Hernandez PA, Peraza JA, Allen KD, Cruz-Almeida Y (2021) Chronic Pain is Associated With Reduced Sympathetic Nervous System Reactivity During Simple and Complex Walking Tasks: Potential Cerebral Mechanisms. Chronic Stress (Thousand Oaks) 5:24705470211030273 34286166 10.1177/24705470211030273 PMC8267022

[3] Bruehl S, Olsen RB, Tronstad C, Sevre K, Burns JW, Schirmer H, Nielsen CS, Stubhaug A, Rosseland LA (2018) Chronic pain-related changes in cardiovascular regulation and impact on comorbid hypertension in a general population: the Tromso study. Pain 159:119–127 28953193 10.1097/j.pain.0000000000001070

[4] Hohenschurz-Schmidt DJ, Calcagnini G, Dipasquale O, Jackson JB, Medina S, O’Daly O, O’Muircheartaigh J, de Lara Rubio A, Williams SCR, McMahon SB, Makovac E, Howard MA (2020) Linking Pain Sensation to the Autonomic Nervous System: The Role of the Anterior Cingulate and Periaqueductal Gray Resting-State Networks. Front Neurosci 14:147 33041747 10.3389/fnins.2020.00147 PMC7527240

[5] Suarez-Roca H, Klinger RY, Podgoreanu MV, Ji RR, Sigurdsson MI, Waldron N, Mathew JP, Maixner W (2019) Contribution of Baroreceptor Function to Pain Perception and Perioperative Outcomes. Anesthesiology 130:634–650 30418212 10.1097/ALN.0000000000002510 PMC6417948

[6] Millan MJ (2002) Descending control of pain. Prog Neurobiol 66:355–474 12034378 10.1016/s0301-0082(02)00009-6

[7] Vanegas H, Schaible HG (2004) Descending control of persistent pain: inhibitory or facilitatory? Brain Res Brain Res Rev 46:295–309 15571771 10.1016/j.brainresrev.2004.07.004

[8] Neugebauer V, Li W, Bird GC, Han JS (2004) The amygdala and persistent pain. Neuroscientist 10:221–234 15155061 10.1177/1073858403261077

[9] Flor H, Diers M, Birbaumer N (2004) Peripheral and electrocortical responses to painful and non-painful stimulation in chronic pain patients, tension headache patients and healthy controls. Neurosci Lett 361:147–150 15135915 10.1016/j.neulet.2003.12.064

[10] Vossen CJ, Vossen HGM, Joosten EA, van Os J, Lousberg R (2015) Does habituation differ in chronic low back pain subjects compared to pain-free controls? A cross-sectional pain rating ERP study reanalyzed with the ERFIA multilevel method. Med (Baltim) 94:e865 10.1097/MD.0000000000000865 PMC4602586 25984683

[11] de Tommaso M, Federici A, Santostasi R, Calabrese R, Vecchio E, Lapadula G, Iannone F, Lamberti P, Livrea P (2011) Laser-evoked potentials habituation in fibromyalgia. J Pain 12:116–124 20685171 10.1016/j.jpain.2010.06.004

[12] Smith BW, Tooley EM, Montague EQ, Robinson AE, Cosper CJ, Mullins PG (2008) Habituation and sensitization to heat and cold pain in women with fibromyalgia and healthy controls. Pain 140:420–428 18947923 10.1016/j.pain.2008.09.018

[13] Montoya P, Sitges C, Garcia-Herrera M, Rodriguez-Cotes A, Izquierdo R, Truyols M, Collado D (2006) Reduced brain habituation to somatosensory stimulation in patients with fibromyalgia. Arthritis Rheum 54:1995–2003 16732548 10.1002/art.21910

[14] Valeriani M, de Tommaso M, Restuccia D, Le Pera D, Guido M, Iannetti GD, Libro G, Truini A, Di Trapani G, Puca F, Tonali P, Cruccu G (2003) Reduced habituation to experimental pain in migraine patients: a CO(2) laser evoked potential study. Pain 105:57–64 14499420 10.1016/s0304-3959(03)00137-4

[15] D’Antono B, Ditto B, Sita A, Miller SB (2000) Cardiopulmonary baroreflex stimulation and blood pressure-related hypoalgesia. Biol Psychol 53:217–231 10967233 10.1016/s0301-0511(00)00044-2

[16] McIntyre D, Kavussanu M, Ring C (2008) Effects of arterial and cardiopulmonary baroreceptor activation on the upper limb nociceptive flexion reflex and electrocutaneous pain in humans. Pain 137:550–555 18037241 10.1016/j.pain.2007.10.018

[17] Randich A, Maixner W (1984) Interactions between cardiovascular and pain regulatory systems. Neurosci Biobehav Rev 8:343–367 6095151 10.1016/0149-7634(84)90057-5

[18] Ditto B, Lewkowski MD, Rainville P, Duncan GH (2009) Effects of cardiopulmonary baroreceptor activation on pain may be moderated by risk for hypertension. Biol Psychol 82:195–197 19646503 10.1016/j.biopsycho.2009.07.009

[19] Ring C, van Veldhuijzen JJ (2007) McIntyre D and Kavussanu M. Hypervolemic hyperalgesia in healthy young adults. J Behav Med 30:449–454 17999172 10.1007/s10865-007-9137-0

[20] Ness TJ, Fillingim RB, Randich A, Backensto EM, Faught E (2000) Low intensity vagal nerve stimulation lowers human thermal pain thresholds. Pain 86:81–85 10779664 10.1016/s0304-3959(00)00237-2

[21] Fardo F, Spironelli C, Angrilli A (2013) Horizontal body position reduces cortical pain-related processing: evidence from late ERPs. PLoS ONE 8:e81964 24278467 10.1371/journal.pone.0081964 PMC3835670

[22] Jacobsen TN, Morgan BJ, Scherrer U, Vissing SF, Lange RA, Johnson N, Ring WS, Rahko PS, Hanson P, Victor RG (1993) Relative contributions of cardiopulmonary and sinoaortic baroreflexes in causing sympathetic activation in the human skeletal muscle circulation during orthostatic stress. Circ Res 73:367–378 8330379 10.1161/01.res.73.2.367

[23] Cooke WH, Ryan KL, Convertino VA (2004) Lower body negative pressure as a model to study progression to acute hemorrhagic shock in humans. J Appl Physiol (1985) 96:1249–1261 15016789 10.1152/japplphysiol.01155.2003

[24] Convertino VA, Cooke WH, Holcomb JB (2006) Arterial pulse pressure and its association with reduced stroke volume during progressive central hypovolemia. J Trauma 61:629–634 16966999 10.1097/01.ta.0000196663.34175.33

[25] Dworkin BR, Filewich RJ, Miller NE, Craigmyle N, Pickering TG (1979) Baroreceptor activation reduces reactivity to noxious stimulation: implications for hypertension. Science 205:1299–1301 472749 10.1126/science.472749

[26] Keller S, Bann CM, Dodd SL, Schein J, Mendoza TR, Cleeland CS (2004) Validity of the brief pain inventory for use in documenting the outcomes of patients with noncancer pain. Clin J Pain 20:309–318 15322437 10.1097/00002508-200409000-00005

[27] Freynhagen R, Baron R, Gockel U, Tolle TR (2006) painDETECT: a new screening questionnaire to identify neuropathic components in patients with back pain. Curr Med Res Opin 22:1911–1920 17022849 10.1185/030079906X132488

[28] Dyson KS, Shoemaker JK, Arbeille P, Hughson RL (2010) Modelflow estimates of cardiac output compared with Doppler ultrasound during acute changes in vascular resistance in women. Exp Physiol 95:561–568 20080867 10.1113/expphysiol.2009.050815

[29] Kinser AM, Sands WA, Stone MH (2009) Reliability and validity of a pressure algometer. J Strength Cond Res 23:312–314 19130648 10.1519/jsc.0b013e31818f051c

[30] Pickering TG, Shimbo D, Haas D (2006) Ambulatory blood-pressure monitoring. N Engl J Med 354:2368–2374 16738273 10.1056/NEJMra060433

[31] Verdecchia P (2000) Prognostic value of ambulatory blood pressure: current evidence and clinical implications. Hypertension 35:844–851 10720605 10.1161/01.hyp.35.3.844

[32] O’Brien E, Mee F, Atkins N, O’Malley K (1991) Accuracy of the SpaceLabs 90207 determined by the British Hypertension Society protocol. J Hypertens 9:573–574 1653299 10.1097/00004872-199106000-00016

[33] Holwerda SW, Luehrs RE, Gremaud AL, Wooldridge NA, Stroud AK, Fiedorowicz JG, Abboud FM, Pierce GL (2018) Relative burst amplitude of muscle sympathetic nerve activity is an indicator of altered sympathetic outflow in chronic anxiety. J Neurophysiol 120:11–22 29537916 10.1152/jn.00064.2018 PMC6093954

[34] Holwerda SW, Luehrs RE, DuBose L, Collins MT, Wooldridge NA, Stroud AK, Fadel PJ, Abboud FM, Pierce GL (2019) Elevated Muscle Sympathetic Nerve Activity Contributes to Central Artery Stiffness in Young and Middle-Age/Older Adults. Hypertension 73:1025–1035 30905199 10.1161/HYPERTENSIONAHA.118.12462 PMC6937199

[35] Norsk P, Ellegaard P, Videbaek R, Stadeager C, Jessen F, Johansen LB, Kristensen MS, Kamegai M, Warberg J, Christensen NJ (1993) Arterial pulse pressure and vasopressin release in humans during lower body negative pressure. Am J Physiol 264:R1024–R1030 8498590 10.1152/ajpregu.1993.264.5.R1024

[36] Holwerda SW, Vianna LC, Restaino RM, Chaudhary K, Young CN, Fadel PJ (2016) Arterial baroreflex control of sympathetic nerve activity and heart rate in patients with type 2 diabetes. Am J Physiol Heart Circ Physiol 311:H1170–H1179 27591221 10.1152/ajpheart.00384.2016 PMC5243209

[37] Dayan L, Hochberg U, Nahman-Averbuch H, Brill S, Ablin JN, Jacob G (2018) Increased Sympathetic Outflow Induces Adaptation to Acute Experimental Pain. Pain Pract 18:322–330 28627793 10.1111/papr.12606

[38] Rennefeld C, Wiech K, Schoell ED, Lorenz J, Bingel U (2010) Habituation to pain: further support for a central component. Pain 148:503–508 20097005 10.1016/j.pain.2009.12.014

[39] Convertino VA, Cooke WH (2002) Relationship between stroke volume and sympathetic nerve activity: new insights about autonomic mechanisms of syncope. J Gravit Physiol 9:P63–P66 15806683

[40] Convertino VA, Ludwig DA, Cooke WH (2004) Stroke volume and sympathetic responses to lower-body negative pressure reveal new insight into circulatory shock in humans. Auton Neurosci 111:127–134 15182742 10.1016/j.autneu.2004.02.007

[41] Sowers JR, Mohanty PK (1987) Effect of advancing age on cardiopulmonary baroreceptor function in hypertensive men. Hypertension 10:274–279 3623680 10.1161/01.hyp.10.3.274

[42] Mark AL, Kerber RE (1982) Augmentation of cardiopulmonary baroreflex control of forearm vascular resistance in borderline hypertension. Hypertension 4:39–46 7061127 10.1161/01.hyp.4.1.39

[43] Price DD, Hu JW, Dubner R, Gracely RH (1977) Peripheral suppression of first pain and central summation of second pain evoked by noxious heat pulses. Pain 3:57–68 876667 10.1016/0304-3959(77)90035-5

[44] Agostinho CM, Scherens A, Richter H, Schaub C, Rolke R, Treede RD, Maier C (2009) Habituation and short-term repeatability of thermal testing in healthy human subjects and patients with chronic non-neuropathic pain. Eur J Pain 13:779–785 19019713 10.1016/j.ejpain.2008.10.002

[45] Chung OY, Bruehl S, Diedrich L, Diedrich A (2008) The impact of blood pressure and baroreflex sensitivity on wind-up. Anesth Analg 107:1018–1025 18713923 10.1213/ane.0b013e31817f8dfe

[46] Fillingim RB, Maixner W, Kincaid S, Silva S (1998) Sex differences in temporal summation but not sensory-discriminative processing of thermal pain. Pain 75:121–127 9539681 10.1016/S0304-3959(97)00214-5

[47] Price DD (1972) Characteristics of second pain and flexion reflexes indicative of prolonged central summation. Exp Neurol 37:371–387 4637957 10.1016/0014-4886(72)90081-7

[48] Edwards L, McIntyre D, Carroll D, Ring C, Martin U (2002) The human nociceptive flexion reflex threshold is higher during systole than diastole. Psychophysiology 39:678–681 12236336 10.1017/S0048577202011770

[49] Edwards L, Ring C, McIntyre D, Carroll D (2001) Modulation of the human nociceptive flexion reflex across the cardiac cycle. Psychophysiology 38:712–718 11446585

